# Estimated Breeding Values for Monetary Earnings and Cutting Points in Brazilian Quarter Horses

**DOI:** 10.3390/ani16213455

**Published:** 2026-11-02

**Authors:** Larissa Y. Rodrigues, Ricardo A. S. Faria, Gustavo H. Alberto, Carolina Zucatelle, Pablo Dominguez-Castaño, Josineudson A. II V. Silva

**Affiliations:** 1 Department of Animal Science, School of Agricultural and Veterinary Sciences (FCAV), São Paulo State University (UNESP), Jaboticabal 14884-900, Brazil; gustavo.alberto@unesp.br; 2 Departament of Animal Breending and Nutrition, School of Veterinary Medicine and Animal Science (FMVZ), São Paulo State University (UNESP), Botucatu 18618-681, Brazil; carolina.zucatelle@unesp.br (C.Z.);; 3 Fonte Boa Innovation Pole, National Institute for Agricultural and Veterinary Research (INIAV, IP), 2005-048 Santarém, Portugal; ricardo.faria@iniav.pt; 4 Department of Animal Biosciences, Swedish University of Agricultural Sciences, SE-750 07 Uppsala, Sweden

**Keywords:** equine, genetic improvement, Bayesian inference, pedigree analysis, earnings, athletic performance, quantitative genetics

## Abstract

In the Brazilian Quarter Horse (QH), cutting points (CP) and monetary earnings (ME) are key success indicators, yet their genetic parameters remain unestablished. This study estimated heritability and breeding values for these traits to guide breeding decisions. Performance data from 1661 QHs competing in official Brazilian cutting events between 1981 and 2020 were analyzed for three traits: total lifetime cutting points (CPt), cutting points at four years of age (CP4), and lifetime monetary earnings (ME). Variance components and genetic parameters were estimated using single-trait Bayesian animal models. Heritability estimates were 0.110 ± 0.038 for CPt, 0.110 ± 0.048 for CP4, and 0.288 ± 0.091 for ME. Although overall genetic trends were positive, in the last decade CPt registered the highest annual genetic gain (0.116). The findings suggest that both CP and ME could be considered as potential selection criteria, and evaluating performance at four years of age (CP4) may favor early genetic progress. While ME shows greater potential for lifelong phenotypes due to its moderate heritability, both traits exhibited comparable selection accuracies; therefore, definitive recommendations require further studies on genetic correlations and expected responses.

## Simple Summary

Selecting the best horses for equestrian sports requires understanding how much of their performance is determined by genetics. In Brazil, Quarter Horses are highly valued for cutting competitions, where horses demonstrate their ability to separate cattle from a herd. However, the genetic basis for their success was not established. In this study, we analyzed historical data from Brazilian cutting Quarter Horses to determine the genetic influence on the points they score and the prize money they receive from the associations. We found that both performance indicators have a genetic foundation, meaning these abilities are passed to offspring. Evaluating horses at four years of age may help accelerate breed improvement by identifying better sires and dams early. Furthermore, both accumulated points and lifetime monetary earnings show potential as valuable tools to guide mating programs. These findings provide a practical first step for breeders to make informed mating decisions based on genetic merit rather than just visual performance, supporting the long-term genetic progress of the cutting Quarter Horse in Brazil.

## 1. Introduction

The Quarter Horse (QH) breed was introduced in Brazil in 1955, with the establishment of the Brazilian Association of Quarter Horse Breeders (ABQM) in 1969 [1], reflecting the breed’s importance in the national scene [2]. The working genetic group were selected based on their usefulness in everyday ranch work, with value placed on individuals who demonstrate cattle-handling skills, responsiveness to commands, and agility in maneuvers, among other traits [3,4,5].

The National Cutting Horse Association (ANCA) of Brazil was founded in 1989 to promote and regulate national cutting competitions, a discipline originated from the daily ranch work [6]. Recent studies reinforce the complexity of this modality, highlighting the high physiological demands placed on these equine athletes [7,8].

According to Hintz [9], the cow sense trait is defined as the horse’s ability to work with cattle efficiently. Similarly, Ellersieck et al. [10] described it as the ability to separate a cow from the herd with minimal assistance from the rider. The cow sense trait is subjective, and cutting competitions are used to evaluate its degree in sports horses, where performance is assessed by one or more judges [6].

The ranking systems of ABQM and ANCA assign points to the top-placed horses in each event and from these records it is possible to generate the cutting points (CP) trait. Furthermore, since both associations offer significant cash prizes and incentives based on age, sex, and lineage, monetary values can be used to derive the monetary earnings (ME) trait. QH breeders frequently base their mating decisions and the choice of breeding stock on the total points and monetary earnings accumulated by the individuals and their offspring, even in the absence of proper genetic evaluation.

Few studies have addressed the estimates of heritability (h^2^) of traits associated with the performance of cutting sport horses, such as the CP and ME traits. Ellersieck et al. [10] reported h^2^ of 0.19 for the cutting scores trait in three-year-old QHs, while the study by Kieffer 1968 [11] found an h^2^ of 0.04 for cutting ability, indicating variability in American populations. While these studies confirm the presence of genetic variability in cutting ability, they focus on subjective scores rather than cumulative performance metrics. Clayton [12] compared the kinematic variables of two groups of cutting horses: elite competitors with monetary earnings exceeding $35,000 and average horses with earnings below this threshold. The study demonstrated that elite horses maintained closer proximity to the mechanical flag at the start, during, and at the end of the run, and exhibited significantly faster reaction times in response to the flag’s movement. The author highlighted the importance of identifying variables with high genetic potential early in a horse’s career, linking kinematic superiority to higher monetary earnings. However, an understanding of how these earnings and accumulated points translate into heritable traits is still lacking in the literature for the Brazilian QH cutting population.

Although genetic parameters for various equine disciplines are documented, genetic studies specifically addressing the cutting QH population remain scarce. Therefore, while breeders frequently rely on CP and ME for empirical mating decisions, the extent to which these performance indicators reflect true additive genetic merit remains largely unexplored. Estimating these parameters is essential to validate whether these traits can be effectively implemented as criteria in genetic improvement programs.

This research aimed to report, for the first time, the parameters and genetic values for the traits cutting points (CP) and monetary earnings (ME) in Brazilian cutting Quarter Horses. We hypothesized that both CP and ME possess sufficient additive genetic variance to be utilized as reliable selection criteria, and that evaluating these traits, especially at an early age, can guide breeding decisions to improve the athletic performance of the population.

## 2. Materials and Methods

The database on CP traits (points each animal earned annually) was obtained from the ABQM website (https://seqm.com.br/ranking/geral; free registration required, accessed on 5 March 2020), while the information on ME traits was collected from the ANCA website (http://www.anca.com.br/, accessed on 25 November 2020). Data were collected from 1661 Quarter Horse athletes in the cutting modality (events registered with ABQM) between 1981 and 2020 in Brazil. The dataset comprised a 40-year historical series that captured the complete foundation and genetic evolution of official cutting competitions in Brazil. Furthermore, given the relatively long generation interval in this equine population [13], genetic parameters and variance components remained biologically stable over recent years, making the estimates robust and directly applicable to current breeding strategies.

The animals were born between 1970 and 2016, and the competition age ranged from 2 to 28 equestrian years. To ensure adequate representation, the eight records from two-year-old animals were merged with the three-year-old class, and 21 animals older than 12 years old were grouped into a single 12-year-old and older class, resulting in 10 age classes.

Records of cutting points (CP) accumulated per year in ABQM events were collected from 1661 animals (627 males). Two CP traits were evaluated from the dataset: Total Cutting Points (CPt) as the sum of cutting points obtained during the sportive life of the animal, and Cutting Points at four years (CP4) as the sum of cutting points accumulated until (and including) four equestrian years of age. After quality control, the CPt trait ranged from 0.5 to 179.25 points per animal (1653, 37.7% males). The CP4 subset comprised 72.2% of the animals with CPt data (452 males) and ranged from 0.5 to 67.25 points per animal. CPt and CP4 were evaluated as a single measure per animal.

The ME trait is the total lifetime prize money earned by an animal, evaluated as a single measure per animal. The ME dataset comprised the same 1653 animals evaluated for CPt, of which 1000 animals (388 males) received monetary prizes in ANCA events. It was assumed that at least one prize was offered per event and all competitors had an opportunity to win.

To account for annual inflation in Brazil, monetary values were adjusted to December 2021 using the General Price Index—Internal Availability (IGP-DI), published by the Getúlio Vargas Foundation (FGV) [14]. These adjusted values were then converted to US dollars ($) to allow for international comparison. To account for non-normal distribution of the ME trait, a base-10 logarithmic transformation (log10) was applied to records of animals with earnings greater than zero (ME > 0) [15,16,17]. To prevent selection bias and accommodate the mathematical undefinedness of log10(0), animals that competed but did not earn monetary prizes (ME = 0) were not subjected to the logarithmic equation and were retained in the dataset with a direct assigned phenotypic value of zero.

The pedigree database was previously described by Rodrigues et al. [13], with genealogical information of animals competing in 2019 and 2020. Consequently, the final pedigree file comprised information on 5479 animals born between 1834 and 2016, with a pedigree information quality of 5.5 ± 1.2 known equivalent generations [18]. Up to seven generations were considered in this study analysis using the PED_DEPTH procedure in RENUMF90 [19]. Database management, consistency check and descriptive analysis were performed using SAS 9.3 [20], while graphics were generated using R software version 4.5.1 [21].

An inherent limitation of this retrospective study was the impossibility to directly evaluate environmental effects such as the class of competition, rider, judges, event location, and regulatory changes, as these detailed historical data were not available. Consequently, to account for these unrecorded sources of variation, the contemporary group (CGs) was defined by the year of first and the year of last record, which allowed group animals that competed in the same general circumstances [17,22]. A total of 241 CGs were formed, containing progeny from 380 different stallions (sires of horses with phenotypes). The number of stallions represented per CG ranged from 1 (76 CGs, 53 different stallions, of which 86% participated in larger CGs) to 35 (1 CG), with an average of 5.54 stallions per CG.

Single-trait analyses were conducted to obtain the variance components and explore the results for the traits CPt, CP4, and ME. The model for the CP4 trait included the systematic effects of CG, equestrian year of birth and sex, the covariate number of years competing until and including four years of equestrian age, and the random effects of animal and residual effects. For CPt and ME, the fixed effects were CG, equestrian year of birth, and the covariates age at last record and number of years competing and the random effects of animal and residual [23,24,25].

The matrix representation of the models was as follows:

y = Xβ + Za + *e*

where y is the vector of observations, β is the vector of systematic effects, a is the vector of direct additive genetic values of the animal, ***e*** is the vector of random residual effects, and X and Z are the incidence matrices that relate the observations to the systematic effects and direct additive genetic random effects, respectively.

Trait analyses were conducted using single Gibbs chains with 1,100,000 cycles and a burn-in period of 100,000 samples, determined after visually verifying the stationary phase of the chain [22]. Estimates were stored every 20 samples, totaling 50,000 samples used to calculate the posterior means, standard deviations, and 95% highest posterior density (HPD95) intervals for the (co)variance components and estimates of heritability (h^2^). Statistical analyses and chain convergence checks were performed using GIBBSF90+ version 3.16 and POSTGIBBSF90 version 3.15 software [19]. Effective sample size (ESS) and effective chain size (ECS) for the three traits were greater than 30 and 200, respectively, indicating proper Gibbs chain convergence for CP4, CPt and ME traits [26,27,28].

Preliminary multi-trait analyses were performed using the same chain length, burn-in period, and sampling parameters as the single-trait models. However, the multi-trait analyses showed inadequate convergence diagnostics, particularly for CP4, with an effective sample size (ESS) of 19.6, and ME, with an effective chain size (ECS) of 96. Consequently, single-trait models were adopted for the final analysis. These models showed improved convergence diagnostics, with ESS values of 495.8, 793.5, and 844.7 and ECS values of 286, 242, and 168 for CP4, CPt, and ME, respectively. Although the single-trait models demonstrated improved sampling efficiency, the ECS for ME remained below the adopted threshold of 200. Therefore, this limitation should be considered when interpreting the genetic parameter estimates for ME. Future studies should explore multi-trait models using longer MCMC chains and additional convergence diagnostics.

The estimates of genetic values (EBV) were obtained using the BLUPF90+ procedure [19] via “OPTION fixed_var mean” command, utilizing the (co)variances components estimated in the present study. Genetic trends were then calculated through the linear regression of the EBVs on the animals’ birth years, with results presented as annual mean trend lines. Accuracy (*ACCi*) was calculated as follows:

$$\mathrm{ACCi}=\sqrt{1-\frac{\mathrm{PEVi}}{\left(1+\mathrm{Fi}\right)\sigma_{a}^{2}}}$$

where *PEVi* is the prediction error variance for each animal, *Fi* is the individual inbreeding coefficient and $\sigma_{a}^{2}$ a is the additive genetic variance.

## 3. Results

**Figure 1:**
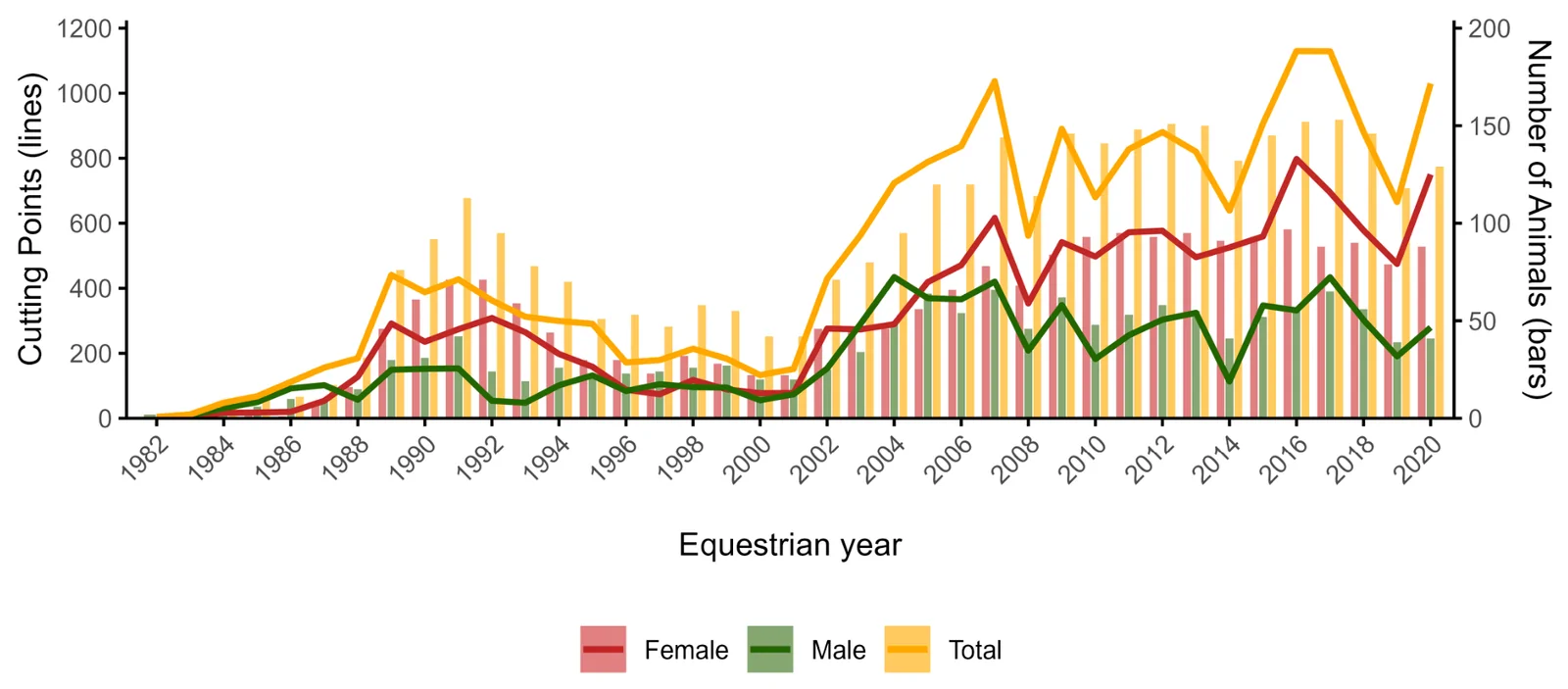
Total number of horses, distributed by sex, and accumulated points of athlete Quarter Horses in Cutting events in Brazil from 1981 to 2020.

Cutting horse competitions were continuously promoted by the ABQM from 1981 to 2020. Of the 1653 animals that participated in the cutting events, 1030 were females (62.3%). The average number of competitors per year was 87, with 1982 and 1983 recording the lowest (2) and 2017 the highest (153) number of competing animals (Figure 1). In the early years of ABQM events, male competitors outnumbered females (Figure 1). Over the 39 years of competitions evaluated, females scored the most points in 29 years (74.3%).

Regarding the CPt trait, females accounted for 62.4% of the overall points accumulated during the study period, averaging 5.90 points per mare, while males accumulated 37.6%, with an average of 5.44 points per male (Table 1). Average points differed significantly between the sexes for CPt (*p* = 0.03) but not for CP4 (*p* > 0.05). The highest individual CPt score observed was 179.25 points for a male and 122.75 points for a female.

Of the 1653 horses earned points (CPt records) in ABQM competitions, 60.5% also earned monetary prizes (ME records) in ANCA-promoted competitions. Across all official competitions, almost US$9.8 million in earnings were distributed to 1000 animals (Table 1). Among the 1030 competing females, 59.4% earned monetary prizes, an average of US$5.9 thousand per awarded mare. For males, 388 out of 623 (62.1%) earned prizes, averaging of US$5.8 thousand. The difference between the average earnings by sex was not significant (*p* = 0.76), indicating no differences between ME means for females and males.

The estimated heritability (h^2^) values for the evaluated traits ranged from low to moderate (Table 2). Heritabilities were low and similar for the trait evaluated at four years of age (CP4) and for total life (CPt). The 95% highest posterior density (HPD95) intervals were widest for CP4 and narrowest for CPt. The ME trait was moderate.

The estimated breeding values (EBVs) indicated a population mean of 0.03 for CPt and 0.18 for CP4 (Table 3), with wide genetic variability observed for CPt when analyzing the minimum and maximum EBV values. Female EBVs were lower than those of males for CPt (−60%) and for CP4 (−23.8%). Based on HPD95, a small number of animals fell below −1.59 points for CPt and above 2.20 points for CP4 (Table 3). For ME, the female mean was 8.3% lower (−0.001 log10) than that of males. Only 5.0% of the evaluated population had EBVs below −0.15 or above 0.23 log10 ME.

**Figure 2:**
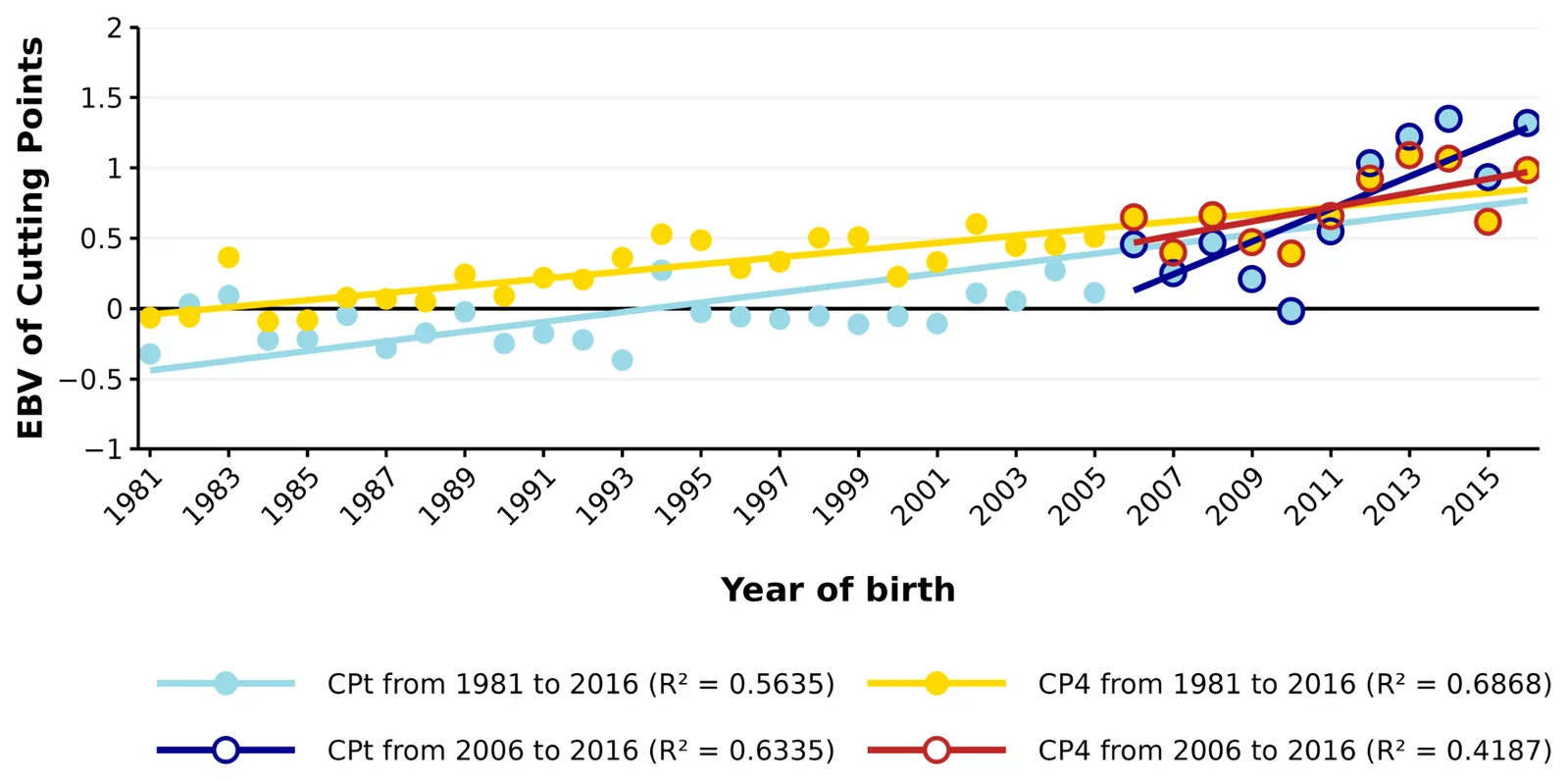
Genetic trends for the cutting points trait in Quarter Horse Cutting athletes in Brazil from 1981 to 2016 and from the last decade of data.

Genetic trends for CPt and CP4 were positive and similar for the total years, showing annual genetic gains of 0.03 and 0.024 points per year, respectively (Figure 2). During the last decade, both traits maintained positive genetic trends, with estimated annual genetic gains of 0.116 points per year for CPt (R^2^ = 0.6335) and 0.050 points per year for CP4 (R^2^ = 0.4187).

**Figure 3:**
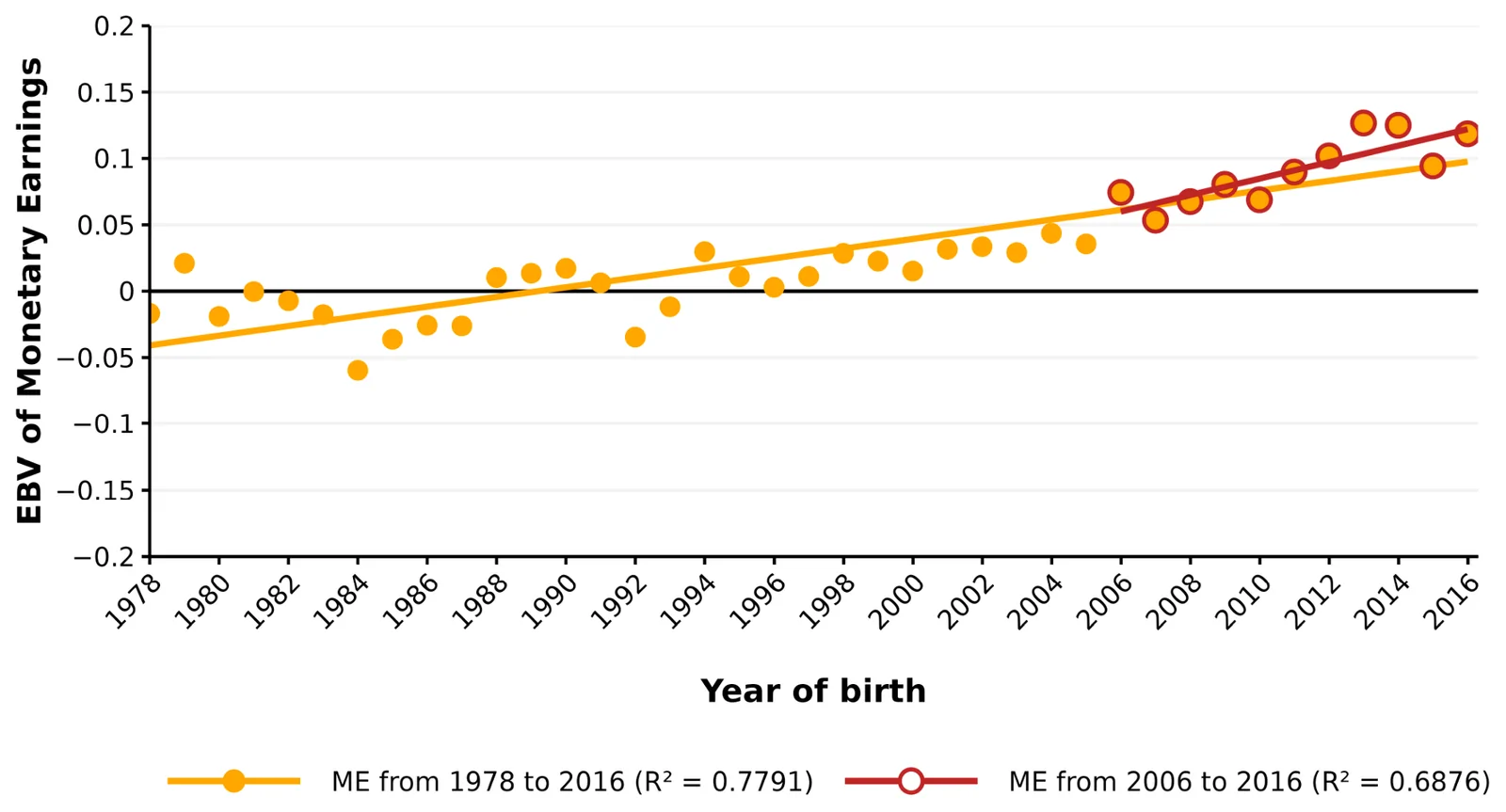
Genetic trends for the monetary earnings trait in Quarter Horse athletes of the cutting modality in Brazil from 1981 to 2016 and from the last decade of data.

The genetic trend for ME was positive over the entire study period, with an estimated annual genetic gain of 0.003 log10 units per year (R^2^ = 0.7791). During the last decade, ME maintained a positive genetic trend, with an estimated annual genetic gain of 0.006 log10 units per year (R^2^ = 0.6876) (Figure 3).

## 4. Discussion

The total number of horses in competitions has consistently increased since the creation of ANCA in 1981 (Figure 1). This growth reflects the successful aim of the association, formed mainly by breeders, competitors, and cutting horse trainers, to promote and consolidate the cutting discipline in Brazil.

The participation of mares in competitions increased alongside the growth and development of the discipline, with females representing the largest number of competing individuals from 1988 onwards. Variation in sex distribution is often driven by management characteristics and animal availability, as observed by Gómez et al. [29] in Eventing and Show Jumping disciplines, where females also outnumber males and geldings. Conversely, Buttram et al. [30] observed prolonged racing longevity in Quarter Horse geldings compared to intact horses, indicating early retirement of mares for reproduction and castration of non-breeding males to extend their athletic careers. Between 1995 and 2002, there was a decline in the number of animals (Figure 1), with similar reports for the Brazilian breeds Mangalarga Marchador, Campolina and Mangalarga in the same period [31,32,33,34] in the same period.

Females accumulated the highest points and monetary earnings in more than half of the evaluated period. This prominence of females is corroborated by studies in other modalities within the breed. Evaluating the profitability of racing QHs, Faria et al. [35] similarly reported a female majority (55.6%), though they found no significant difference in the average earnings between sexes, which is consistent with the findings of the present study. Furthermore, Santana et al. [36] reported similar high female participation in Brazilian barrel racing and found that females significantly outperformed males. These findings collectively suggest that Brazilian QHs prioritize testing females across various disciplines, maintaining this selection strategy even when average performance metrics between sexes do not significantly differ.

To the best of our knowledge, the present study is the first report on cutting points trait for the QM breed in Brazil. The literature addressing cow sense and the CP trait is scarce and outdated. Ellersieck et al. [10], evaluating 3399 cutting horse athletes in the NCHA Futurity category (exclusively for three-year-old horses), found h^2^ values for the cutting scores trait that were higher than those in the present study (0.19 ± 0.05). Kieffer 1968 [11], evaluating 92 paternal half-siblings (offspring of 34 sires), described h^2^ values for the cutting ability trait that were lower than those in the present study (0.04). The authors of both studies suggested they were working with pre-selected data. Because the animals had been previously selected for the discipline and came from similar lineages, the variation and resulting heritability estimates were likely reduced. Similarly, the present study also relies on a pre-selected population, as historical data availability from the associations is restricted exclusively to horses that successfully competed and earned points. Therefore, the present results reflect the reality of the awarded cutting population and should be interpreted within this specific context, avoiding extrapolation to the broader Quarter Horse breed. Additionally, as the CP trait is evaluated subjectively, future studies should analyze the individual components of the final score given by the judges to identify those with the greatest genetic importance.

Multi-trait models applied to historical and sparse datasets, particularly those containing skewed and zero-inflated traits like ME and CP4, frequently suffer from a lack of parameter identifiability and failure to reach convergence in Bayesian samplers. In our preliminary analyses, multi-trait Gibbs chains failed to achieve the minimum effective chain size and stationary phase required for valid inferences. Consequently, the single-trait approach was computationally required to obtain reliable variance components for this Quarter Horse population.

The heritability estimate observed for the CP4 trait (Table 2) indicates its viability as an early selection criterion. Although the magnitude of h^2^ for CP4 is comparable to that of total lifetime performance (CPt), selecting animals at four years of age is advantageous. The effectiveness of this strategy is well-supported by studies in other sport horse breeds, which demonstrate that performance evaluated at four years of age exhibits positive genetic correlations with lifetime results [37,38,39]. Consequently, early evaluations and progeny testing have proven reliable for the selection of young sires and dams before their sporting careers are fully concluded [39,40]. Previous pedigree analysis of this specific population revealed a prolonged average generation interval of 11.7 ± 8.8 years [13]. Early selection can counteract this delay by reducing the generation interval. Therefore, identifying superior sires and dams at a younger age directly accelerates the annual genetic gain in the cutting horse population.

As cited by Langlois [41], acts as a key performance indicator in equines, representing the sum of prizes obtained over an animal’s sporting life, and along with other traits, demonstrate variation and heritability [42]. Although genetic parameters for ME have not been previously published for cutting Quarter Horses (QHs), studies evaluating the Brazilian racing QH line reported h^2^ estimates ranging from 0.10 to 0.33, depending on the age of evaluation and the statistical model [35,43]. Furthermore, similar moderate heritability values have been reported for monetary earnings across other major sport horse breeds globally. In Thoroughbreds, heritability estimates for lifetime or cumulative earnings range from 0.11 to 0.25 [16,44], while in Arabian racing horses, estimates for earnings vary from 0.14 to 0.46 [15,45]. The heritability estimate of 0.28 for ME observed in the present study (Table 2) is highly consistent with the range reported for QHs in Brazil, indicating that this trait possesses sufficient additive genetic variance for inclusion in cutting QH breeding programs.

The heritability estimates found in this study reflect the complex and demanding nature of the cutting competitions. As highlighted by Sullivan et al. [8], young cutting horses are exposed to high physiological stress due to training and competition routine, often leading to early ligament injuries. This implies that a significant portion of the phenotypic variance is influenced by environmental factors such as training intensity and veterinary care. Consequently, identifying and selecting animals with higher EBVs for cutting points and earnings not only improves athletic performance, but may also indirectly select individuals with greater physical resilience.

Furthermore, decreased performance in cutting horses is frequently associated with underlying musculoskeletal issues. Swor et al. [46] reported that most cutting horses examined in their study presented with a decline in athletic ability rather than clear lameness. This subtle physical deterioration directly impacts a horse’s capacity to maintain high scores and accumulate earnings over its lifetime. Therefore, future genetic evaluations in the QH breed should investigate traits related to sports longevity and stayability, aiming to identify breeding stock capable of sustaining high performance while minimizing the risk of career-ending injuries.

Although specific repeatability estimates were not evaluated in the present study, the concepts of performance consistency over time are crucial for understanding the genetic parameters of cutting horses. In other Quarter Horse disciplines, performance traits often exhibit moderate to high consistency, with parameters ranging from 0.28 to 0.46 [36,43,47]. In cutting horses, however, the low heritability estimates observed for cutting points (0.110 for both CPt and CP4) and the modest EBV accuracies (averaging 0.20 to 0.23) suggest a influence of non-genetic factors. A primary explanation for this variation is the extended sporting longevity characteristic of the cutting discipline. Unlike racing horses, which typically have short careers, cutting horses often remain active to more advanced ages. This aligns with the observations of Santana et al. [36], who noted that across long age trajectories, distinct permanent environmental factors significantly influence performance at different stages of life. In cutting horses, this prolonged athletic lifespan inevitably leads to performance fluctuations. The accumulated physical wear and subclinical musculoskeletal issues make it inherently difficult to maintain peak performance over the years. Therefore, these extensive non-genetic influences likely inflate the environmental variance, thereby diluting the proportion of additive genetic variance observed in the phenotype over the animal’s lifetime. This highlights the biological and physical toll of long-term competitions and reinforces the importance of age-specific evaluations, such as CP4, to more accurately capture the animal’s genetic potential.

The estimated breeding values (EBVs) of the CP and ME traits (Table 3) indicate that there is variability within the population in the traits and between sexes. Selecting animals with the highest genetic merit (EBVs) as breeders will enable continuous genetic gain for these traits in future progeny.

Notably, males exhibited higher EBVs than females for all traits (Table 3). This dynamic differs from that of racing QHs, where Faria et al. [43] observed that breeding females often presented genetic equality or superiority compared to stallions, potentially due to the dilution of genetic gain caused by a higher number of offspring per sire. In the cutting population, the observed genetic superiority of males indicates that the use of reproductive biotechnologies, such as artificial insemination with frozen semen, can enable the dissemination of superior genetics from young stallions while they are still competing. This strategy aligns with the need to reduce the generation interval and maximize annual genetic gain.

Although the genetic trends for both the CPt and CP4 traits exhibited a positive direction over the evaluated period (Figure 2), the magnitude of annual genetic gains given by the slope coefficients are nearly zero. This slow rate of genetic progress may reflect the challenges of the reduced effective population size previously identified in this specific population by Rodrigues et al. [13]. Similar slow genetic progress trends were documented by Faria et al. [35,43] in the Brazilian racing QH population, where a narrow genetic base ultimately restricted long-term gains. Vilela et al. [48] reported the detrimental effects of a narrowed genetic base on athletic performance in Peruvian Paso horses, indicating that inbreeding depression significantly impairs key functional and biomechanical traits in this breed. Therefore, to sustain and maximize genetic gains in the cutting QH population, breeders should consider the use of breeding animals with superior EBVs and accuracies for cutting point traits, while continuously monitoring inbreeding.

Similarly, the genetic trend for ME (Figure 3) demonstrated overall genetic progress across the 44 years of birth evaluated. It is crucial to note that the reduced variation observed is partially a statistical consequence of the log10 transformation applied to normalize the monetary data [9,15,49], which inherently compresses the scale of the estimated breeding values. Furthermore, while the CP evaluation relies on a predetermined, standardized scoring scale, ME may be more susceptible to environmental and macroeconomic factors, such as the number of active competitors (Figure 1), association investments, and available sponsorships in any given year.

When analyzing only the last decade of available data, the ME maintained a positive trend (Figure 3). This trajectory differs from the pattern reported by Faria et al. [43] for racing QHs, where genetic trends decreased over a similar 10-year period (2005 to 2014). Even so, the annual genetic gains for ME, considering both the entire evaluated period and the last decade, were the lowest compared to CPt and CP4. Considering the moderate heritability of ME compared to the low estimates for CPt and CP4, this modest genetic progress suggests unexploited potential. Therefore, breeders should prioritize the strategic mating of individuals with superior EBVs and accuracy for ME.

Beyond mitigating inbreeding and addressing the recent slowdown in genetic progress, carefully planned mating strategies are crucial in cutting QHs due to recessive genetic disorders. A primary concern is Hereditary Equine Regional Dermal Asthenia (HERDA), a severe skin condition prevalent in the cutting QH [50], presenting a 5.8% carrier frequency in the Brazilian QH population [51]. Because carriers of the defective allele exhibit outstanding athletic performance and monetary earnings [52,53], strictly phenotypic selection without genotypic monitoring can increase the disease’s frequency. Therefore, breeding programs should integrate the estimated breeding values for ME and CP with genetic testing for known diseases. This combined approach helps optimize genetic gain and profitability while preventing carrier-to-carrier matings, ensuring the long-term health and sustainability of the cutting QH population.

Finally, ongoing collaboration among the breed association, trainers, and breeders in providing more information regarding the uses of cutting athlete horses after they conclude their sports careers will be vital. Such information can yield important insights for maintaining genetic variability and promoting the continued genetic evolution of this Quarter Horse population in Brazil.

## 5. Conclusions

The results indicated that cutting points traits exhibited low heritability estimates in Brazilian Quarter Horses, suggesting that genetic selection for these traits may contribute to long-term improvement. Performance recorded at four years of age may provide useful information for earlier genetic evaluation. In contrast, monetary earnings demonstrated a moderate heritability estimate, emerging as a promising trait to improve the lifetime performance of this population. However, the relative advantages of the evaluated traits for selection require further investigation, particularly regarding genetic correlations and expected selection response. The observed genetic trends should be interpreted cautiously, considering the historical structure of the dataset and potential environmental influences on competition performance and monetary earnings.

## Figures and Tables

**Table 1:** Statistical description of the total and mean values for cutting points (CPt and CP4) and monetary earnings (ME) in Brazilian Quarter Horses.

Type of Records	Trait		
	Cutting Points		Monetary Earnings (U$)
	Total	4 Years	Total
Males (µ ± sd)	7301.5 (11.7 ± 17.2)	2987.25 (6.6 ± 7.9)	3,651,776.23 (9436.11 ± 11,646.16)
Females (µ ± sd)	12,259.75 (11.9 ± 15.7)	5143.0 (6.9 ± 7.5)	6,140,404.00 (10,033.34 ± 11,808.54)
Total (µ ± sd)	19,561.25 (11.8 ± 16.3)	8130.25 (6.8 ± 7.6)	9,792,180.17 (9801.98 ± 11,743.66)

µ, mean per animal; sd, standard deviation; U$, United States dollar.

**Table 2:** Estimates of heritability of cutting points and monetary earnings for performance at 4 years old and total life of cutting Quarter Horses in Brazil.

Trait	Heritability			
	Mean ± sd	Median	Mode	HPD95
CP4	0.110 ± 0.048	0.107	0.108	0.012 to 0.200
CPt	0.110 ± 0.038	0.107	0.102	0.038 to 0.187
ME	0.288 ± 0.091	0.285	0.295	0.115 to 0.472

sd, standard deviation; HPD95, 95% highest posterior density; CPt, sum of lifetime cutting points; CP4, cutting points at 4 years old; ME, lifetime monetary earnings.

**Table 3:** Descriptive statistics of estimated breeding values of Cutting Points (CP) and Monetary Earnings (ME) traits in the Quarter Horse breed in Brazil.

	Estimated Breeding Values			Accuracy	
	Pedigree			Pedigree	Breeding Animals
	Mean ± sd	Minimum	Maximum	Mean ± sd	Mean ± sd
CP4	0.18 ± 0.68	−1.75	7.29	0.20 ± 0.16	0.31 ± 0.12
Males	0.21 ± 0.75	−1.75	7.29	0.21 ± 0.17	0.35 ± 0.14
Females	0.16 ± 0.64	−1.46	5.98	0.20 ± 0.15	0.29 ± 0.11
CPt	0.03 ± 0.96	−4.30	15.47	0.23 ± 0.17	0.32 ± 0.14
Males	0.05 ± 1.02	−4.30	15.47	0.24 ± 0.18	0.37 ± 0.15
Females	0.02 ± 0.93	−4.03	10.88	0.23 ± 0.16	0.30 ± 0.13
ME (log10)	0.011 ± 0.088	−0.629	0.551	0.23 ± 0.22	0.33 ± 0.20
Males	0.012 ± 0.093	−0.337	0.551	0.24 ± 0.24	0.38 ± 0.22
Females	0.011 ± 0.086	−0.629	0.520	0.23 ± 0.21	0.31 ± 0.19

sd, standard deviation; CPt, sum of lifetime cutting points; CP4, cutting points at 4 years old; ME, lifetime monetary earnings.

## Data Availability

The data presented in this study are not publicly available for privacy reasons, as they belong to the Brazilian Association of Quarter Horse Breeders (ABQM) and the National Cutting Horse Association (ANCA).
